# Prediction Models for Postoperative Delirium Among Cancer Patients: A Scoping Review

**DOI:** 10.3390/healthcare14142207

**Published:** 2026-07-21

**Authors:** Chaoqun Ma, Yao Wu, Jiawen He, Jialian Huang, Yingying Li

**Affiliations:** School of Nursing, Guangdong Pharmaceutical University, Guangzhou 510310, China; 2112376015@stu.gdpu.edu.cn (C.M.); 2112376021@stu.gdpu.edu.cn (Y.W.); 2112419009@stu.gdpu.edu.cn (J.H.)

**Keywords:** postoperative delirium, cancer, prediction model, scoping review, risk assessment

## Abstract

**Highlights:**

**What are the main findings?**

- POD incidence varies according to cancer type, surgery type, assessment tool, and assessment timing.
- Logistic regression remains the most commonly used modeling method while some machine-learning approaches show favorable discrimination. However, most models lack external validation and clinical utility assessment.

**What are the implications of the main findings?**

- Stratified models and standardized POD assessment criteria are needed to address heterogeneity in POD incidence.
- Transparent reporting of machine-learning methods and standardized validation may improve model reliability, interpretability, and clinical applicability.

**Abstract:**

**Objectives**: To systematically map postoperative delirium (POD) prediction models in cancer patients, focusing on study design, modeling methods, performance evaluation, and reporting standards. **Methods**: Following the Arksey and O’Malley framework and PRISMA-ScR guidelines, nine databases were searched from inception to 24 April 2026. Studies developing or validating POD prediction models in cancer patients were included and narratively synthesized. **Results**: Thirty-two studies conducted in China, South Korea, the United States, the Netherlands, and Japan were included, most of which had a high risk of bias. POD incidence ranged from 6.70% to 46.39%. The Confusion Assessment Method was the most common assessment tool, and logistic regression was the predominant modeling approach. The most frequently identified predictor domains were age, operation/anesthesia time, preoperative nutritional indicators, preoperative inflammatory indicators, and American Society of Anesthesiologists physical status classification. AUC values ranged from 0.690 to 0.973, and C-index values ranged from 0.783 to 0.963. However, 43.75% of studies only evaluated performance in the development dataset, 25.00% conducted external validation, and 43.75% did not assess clinical utility. Although 87.50% of studies visually presented models, interpretability analyses for machine-learning models were insufficient. **Conclusions**: Existing POD prediction models for cancer patients show promising discrimination, but their readiness for routine clinical use remains limited by insufficient external validation, inconsistent clinical utility assessment, and incomplete model transparency. These models should currently be regarded as risk stratification tools rather than definitive clinical decision aids. Future studies should prioritize standardized reporting, multicenter external validation, model updating, and decision-curve analysis to support safe clinical translation.

## 1. Introduction

Cancer has become a critical global public health challenge. In 2023, there were 18.5 million new cancer cases and 10.4 million cancer-related deaths worldwide [1]. A variety of therapeutic options are currently available for malignant tumors, including radiotherapy, chemotherapy, cell therapy, and gene therapy. Nevertheless, surgical resection remains the primary curative treatment for most cancers [2]. A large proportion of cancer patients require one or more surgical procedures as a part of their oncological management. Despite its indispensable clinical value, surgical intervention is associated with a variety of adverse perioperative complications and physiological disturbances. Among these conditions, postoperative delirium (POD) is of particular clinical concern because of its detrimental effects on cognitive function and health-related quality of life.

POD is an acute, fluctuating neurocognitive disorder characterized by impaired consciousness, inattention, disorientation, and altered cognitive function. It typically emerges during anesthesia recovery or within 2–5 days after surgery [3]. Compared with general surgical populations, patients with malignant tumors are particularly susceptible to POD due to factors such as advanced age, chronic inflammatory stress, malnutrition, multiple comorbidities, extensive surgical trauma, prolonged anesthesia exposure, and concurrent chemotherapy or immunotherapy [4]. The development of POD not only increases postoperative morbidity, prolongs hospital stay, raises medical costs, and elevates the risk of falls and mortality, but also contributes to long-term cognitive decline, impaired quality of life, and poor oncological prognosis in cancer survivors [5,6,7]. Early identification of high-risk individuals and targeted perioperative preventive interventions are therefore essential for reducing POD-related adverse outcomes.

Prediction models integrate current and historical clinical data to quantify an individual’s risk of subsequent clinical outcomes, enabling clinicians to deliver personalized monitoring and preventive interventions prior to symptom onset [8]. In recent years, a growing body of research has constructed POD prediction models for cancer patients using traditional statistical methods such as logistic regression (LR), as well as machine-learning algorithms including random forest (RF), support vector machine (SVM), and extreme gradient boosting (XGBoost) [9,10]. Predictors incorporated into existing models cover demographic characteristics [11,12], cognitive function [13,14], inflammatory and nutritional biomarkers such as the neutrophil-to-lymphocyte ratio (NLR), Controlling Nutritional Status (CONUT), and systemic immune-inflammation index (SII) [15,16], surgical and anesthetic variables [10,17], psychological factors [12], and others.

Existing studies have investigated the application of POD prediction models in cancer patients. However, such models differ across cancer types, study populations, and different sample sizes, using different selected predictors, modeling algorithms, and validation strategies, with varying predictive performance. The methodological quality, reporting standards, and clinical applicability of these models also remain inconsistent. To date, systematic synthesis of POD prediction models in cancer patients remains limited. Therefore, this study summarizes and compares the predictive performance of existing POD prediction models and identifies core predictive indicators, aiming to provide evidence-based support for clinical decision-making and to inform future research.

## 2. Materials and Methods

### 2.1. Study Design

This scoping review adopted the Arksey and O’Malley methodological framework [18], which outlines a six-stage process: defining the research question, identifying relevant studies, selecting eligible studies, extracting data, synthesizing findings, and reporting results. The review was reported in accordance with the Preferred Reporting Items for Systematic Reviews and Meta-Analyses Extension for Scoping Reviews (PRISMA-ScR) guidelines [19]. The protocol was pre-registered on the Open Science Framework (https://osf.io, accessed 23 April 2026; registration DOI: 10.17605/OSF.IO/SKCA9).

### 2.2. Research Questions

(1) What types of prediction models have been developed for POD in cancer patients, and what is their overall predictive performance?
(2) What construction methodologies and study sample characteristics are reported in the development of these models?
(3) What core predictors and variables are incorporated into POD prediction models for cancer patients?
(4) What are the key limitations of current models, and what directions should future research take?

### 2.3. Identifying Relevant Studies

We searched nine electronic databases (CINAHL, Embase, PubMed, PsycINFO, Web of Science, CNKI, WanFang Data, VIP, and CBM) from inception to 24 April 2026. The search strategy combined Medical Subject Headings (MeSH) and free words, with the core search terms including “cancer”, “postoperative delirium”, and “risk prediction model”. These terms were adjusted appropriately according to the controlled vocabulary and search rules of different databases to ensure comprehensive retrieval. The retrieval was limited to original research published in English and Chinese. Additionally, the references of the included studies were manually searched to identify additional relevant studies. The full electronic search strategy for each database is provided in Table S1.

### 2.4. Study Selection

The Population–Concept–Context (PCC) framework [20] was applied to define the eligibility criteria for this scoping review. The Population comprised adult cancer patients aged ≥ 18 years who underwent surgical treatment. The Concept focused on POD prediction models, defined broadly to include any model developed or validated to predict the risk of delirium after surgery in cancer patients. The Context encompassed studies examining the construction, validation, performance, or application of POD prediction models in cancer patients who received surgical intervention.

#### 2.4.1. Inclusion Criteria

(1) Adult cancer patients aged ≥ 18 years who underwent surgical treatment; (2) POD prediction model for cancer patients, with a clear definition of POD as an acute confusional state occurring within 24 h to 7 days after surgery [21], diagnosed using validated tools such as the Confusion Assessment Method (CAM), Confusion Assessment Method for the Intensive Care Unit (CAM-ICU), 3-Minute Diagnostic Interview for CAM-defined Delirium (3D-CAM), Four-item Acute Delirium Test (4AT), or Diagnostic and Statistical Manual of Mental Disorders (DSM); (3) the study involved the construction, validation, or evaluation of POD prediction models; (4) original research design for model development or validation, including cross-sectional, cohort, and case–control studies; and (5) English or Chinese publications with accessible full text.

#### 2.4.2. Exclusion Criteria

(1) Animal studies, reviews, systematic reviews, meta-analyses, study protocols, conference abstracts, or commentaries (non-primary research); (2) studies that focused exclusively on patients younger than 18 years, non-cancer patients, or mixed populations in which data for adult cancer patients could not be separately extracted; (3) studies focusing on prognostic models for POD (rather than prediction models); (4) models that included only a single predictor, test, or marker; and (5) studies with unavailable full text or incomplete data that could not be used for analysis.

### 2.5. Data Extraction

EndNote 20 software was used to remove duplicate records from the retrieved records. Two independent researchers (A1 and A2) independently conducted the preliminary screening of titles and abstracts, followed by full-text evaluation in accordance with predefined inclusion and exclusion criteria to determine, so as to determine the final included studies. Any discrepancies arising during the screening process were resolved through consensus discussion with a third senior researcher (A5). A standardized data extraction form was developed based on the CHARMS checklist for systematic reviews of prediction modeling studies [22]. Extracted variables included publication year, study region, research population, data collection method, sample size, and incidence of POD. In addition, key reporting characteristics related to prediction model development and validation were extracted according to Transparent Reporting of a multivariable prediction model for Individual Prognosis Or Diagnosis (TRIPOD)-relevant items, including POD assessment method and assessment window, candidate predictor definition, number of POD events, missing data handling, predictor selection strategy, complete model specification, internal validation, external validation, calibration assessment, clinical utility evaluation, and model presentation or availability. For studies using machine-learning methods, additional information was extracted, including the algorithms used, sample size, number of POD events or POD incidence, class imbalance handling, feature selection strategy, validation method, reported performance metrics, calibration assessment, clinical utility evaluation, and model interpretability methods. During data extraction, potential cohort overlap or data reuse was assessed by comparing authorship, institutions, study periods, sample sizes, cancer types, surgical procedures, clinical trial registration numbers, data sources, and cohort descriptions. Studies with clear or possible overlap were not excluded automatically, but were flagged and interpreted cautiously.

### 2.6. Bias Risk Assessment

This study used the Prediction model Risk Of Bias ASsessment Tool (PROBAST) [23] to assess the risk of bias and applicability of the included prediction modeling studies. The tool consists of four evaluation domains: participants, predictors, outcomes, and statistical analysis. Each domain was independently rated as having low, high, or unclear risk of bias. The participant domain evaluated population representativeness and selection bias; the predictor domain assessed the accuracy and standardization of variable measurements; the outcome domain examined the clarity and consistency of outcome definitions and diagnostic criteria; and the statistical analysis domain reviewed the appropriateness of analytical methods and issues such as overfitting. All assessments were conducted independently by two researchers (A3 and A4), and discrepancies were resolved through discussion or consultation with a third reviewer (A5).

## 3. Results

### 3.1. Overview of the Studies

**Figure 1:**
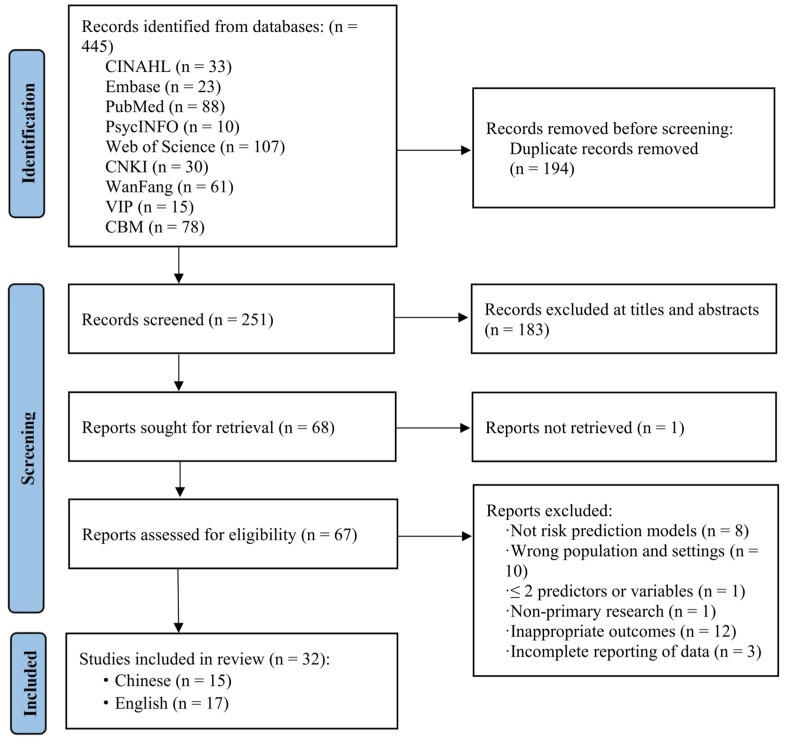
PRISMA flow chart.

After the initial database search, a total of 445 relevant records were retrieved. Ultimately, 32 studies were included in this review [9,10,11,12,13,14,15,16,17,24,25,26,27,28,29,30,31,32,33,34,35,36,37,38,39,40,41,42,43,44,45,46], among which 17 were published in English and 15 in Chinese. The detailed study screening process is presented in Figure 1.

### 3.2. Basic Characteristics of Included Studies

Among the included studies, 29 were published between 2022 and 2026, and 3 were published between 2017 and 2021. In terms of geographical distribution, 28 studies (87.50%) were conducted in China, while the remaining studies were conducted in South Korea, the United States, the Netherlands, and Japan. This indicates that the current evidence base was geographically concentrated, with limited representation from other healthcare systems and populations. Regarding study design, 59.38% of the studies adopted a retrospective design, and all included studies were cohort studies except for one cross-sectional study. Although the cross-sectional study had a high risk of bias and could not support temporal inference, it was retained because it provided supplementary information on the current status and risk factor distribution of the target population. This is consistent with the aim of a scoping review to comprehensively map the available evidence in the field. The basic characteristics of the eligible studies are summarized in Table 1. Potential cohort overlap or data reuse among studies from the same or related research groups was further assessed and is summarized in Table S2. One pair of studies was judged to have clear or highly likely cohort overlap, one pair was judged to have possible partial overlap, and one study used public and synthetic data with unclear independence of the original dataset. These studies were retained because they addressed different modeling or analytical aims, but their findings were interpreted cautiously rather than treated as fully independent evidence.

### 3.3. Incidence of POD and Related Assessment Tools

In total, 18,253 participants were included across the 32 studies. Reported POD incidence ranged from 6.70% to 46.39%, with 10 studies (10/32, 31.25%) reporting an incidence rate greater than 20.00% (Table 1). There was substantial variation in cancer types and POD assessment methods across the included studies, which may be an important factor contributing to the differences in the reported incidence of POD.

Various POD diagnostic criteria and assessment tools were used in the included studies. The CAM series (CAM, CAM-ICU, 3D-CAM) was the most frequently applied, which was used in 24 studies (75.00%). Diagnostic criteria based on DSM-IV/DSM-V were adopted in 7 studies (21.88%). The Four-item Acute Delirium Test (4AT) was utilized in one study (3.13%). In addition, 3 studies adopted combined assessment tools or diagnostic standards (9.38%) (Table S3).

### 3.4. Risk of Bias and Applicability Assessment

Risk of bias was common among the included studies. According to the PROBAST assessment, high risk of bias was identified in 24 studies (75.00%) in the analysis domain, making this domain the major contributor to the overall risk of bias. The most frequent methodological limitations were insufficient sample size relative to the number of candidate predictors or POD events, failure to meet the commonly recommended threshold of at least 10 events per predictor variable, reliance on univariable analysis for predictor screening, and inadequate assessment or correction of model overfitting and optimism. According to PROBAST guidance, inadequate sample size, inappropriate predictor selection, poor handling of missing data, and insufficient assessment of overfitting or optimism are important sources of bias in prediction model studies [47]. In the present review, these issues were frequently observed in the analysis domain and therefore contributed to the overall high risk of bias.

Applicability concerns were also identified in part of the evidence base. Sixteen studies were rated as having high or unclear concerns regarding applicability, including 15 studies with high concerns and 1 study with unclear concerns. These concerns were mainly related to restricted study populations, single-center data sources, heterogeneous cancer types or surgical procedures, and insufficient evidence of model transportability. Although some models reported acceptable discrimination in the original development datasets, the limited use of external validation reduced confidence in their generalizability to other perioperative oncology settings. In addition, incomplete reporting of key analytical procedures, such as missing data handling, calibration assessment, and model validation, may have limited the interpretability and reproducibility of several models. Detailed results of the PROBAST assessment are presented in Table 2.

### 3.5. Overview of Model Types and Predictive Performance

We summarized the cancer populations, modeling approaches, validation strategies, and overall predictive performance of the included POD prediction models. Most models were developed using traditional regression-based approaches, particularly logistic regression, whereas a smaller proportion combined logistic regression with machine-learning algorithms. Overall, the reported discrimination ranged from moderate to excellent. However, calibration assessment, clinical utility evaluation, external validation, and transparent reporting were not consistently performed across studies.

#### 3.5.1. Study Population and Sample Characteristics

Lung cancer and colorectal cancer were the most common cancer types, with 5 studies each. Esophageal cancer, oral cancer, and gastric cancer were covered in 3 studies each, while head and neck cancer as well as prostate cancer were each explored in 2 studies. Other tumor types, including urological tumors, gastrointestinal tumors, laryngeal cancer, glioblastoma, abdominal malignant tumors, gynecologic cancers, hepatocellular carcinoma, and cervical cancer, were each addressed in 1 study. The sample sizes of the included studies ranged from 106 to 2570, and 53.12% of the studies included more than 500 patients.

#### 3.5.2. Modeling Methods and Validation Strategies

Among the 32 included studies, a total of 58 prediction models were developed. At the study level, and two main modeling approaches were identified: logistic regression-based models, and models combining traditional regression with machine-learning algorithms. Logistic regression alone was used in 28 studies, while the remaining 4 studies combined logistic regression with machine-learning algorithms for model construction or comparison. Regarding model validation, the most frequently used method was external validation (8 studies), followed by random split (7 studies), bootstrap method (6 studies), and cross-validation (5 studies). Some studies adopted multiple validation strategies simultaneously.

#### 3.5.3. Overall Model Performance

Model performance was evaluated in three domains: discrimination, calibration, and clinical utility. In terms of discrimination, 23 studies only reported the area under the curve (AUC), 4 only reported the C-index, and 5 reported both metrics. The AUC values of the included POD prediction models ranged from 0.690 to 0.973, while the C-index values ranged from 0.783 to 0.963, indicating moderate to excellent discriminatory performance. For calibration assessment, 8 studies only used calibration curves, 2 only used the Brier score, and 6 only applied the Hosmer–Lemeshow test. Another 6 studies combined the Hosmer–Lemeshow test with calibration curves, and 1 study used all three calibration approaches. Among the 13 studies performing the Hosmer–Lemeshow test, all reported non-significant *p* values (0.090–0.990). Brier scores ranged from 0.07 to 0.115 in the 3 studies, collectively suggesting good consistency between predicted and observed risks. Clinical utility was evaluated using decision curve analysis (DCA) in 15 studies, which suggested favorable net clinical benefits across a wide range of threshold probabilities. Taken together, existing POD prediction models in cancer patients showed generally acceptable to excellent discrimination, but the interpretation of their clinical applicability remains limited by inconsistent external validation, incomplete calibration reporting, and insufficient assessment of clinical utility.

#### 3.5.4. Comparative Summary of Machine-Learning-Based Models

A comparative summary of machine-learning-based POD prediction models is presented in Table S4. Among the 32 included studies, four studies used machine-learning or artificial intelligence algorithms for model construction or comparison. The algorithms included classification and regression tree (CART), random forest, support vector machine, extreme gradient boosting, artificial neural network, Bayesian network, gradient boosting machine, K-nearest neighbors, AdaBoost, LightGBM, CatBoost, linear support vector machine, multilayer perceptron, and Gaussian Naive Bayes.

Model performance was mainly reported using AUC or AUROC. Shen and Xu [24] reported that the CART model achieved an AUC of 0.837. Weng et al. [34] found that random forest showed the best performance, with AUC values of 0.889 in the development cohort and 0.882 in the validation cohort. Wan et al. [10] compared ten machine-learning models, obtaining AUROC values ranging from 0.708 to 0.802. Zhu et al. [9] compared multiple artificial intelligence algorithms, and linear support vector machine achieved the highest AUC of 0.763. However, accuracy, sensitivity, specificity, recall, F1-score, calibration, and clinical utility were not consistently reported across all machine-learning studies.

Overall, although several machine-learning models showed favorable discrimination, direct ranking of algorithms was not appropriate because of heterogeneity in cancer type, dataset characteristics, feature selection methods, validation strategies, and reported performance metrics. In addition, class imbalance handling, external validation, and interpretability analyses remained insufficiently reported in some machine-learning-based studies.

#### 3.5.5. Reporting Completeness and Transparency

Reporting completeness was assessed using selected TRIPOD-relevant items, and the detailed results are presented in Table S5. Overall, reporting of outcome assessment was relatively complete. Most studies clearly reported the POD assessment method, POD assessment window, number of POD events, and predictor selection strategy. However, reporting was less complete for several key items related to model reproducibility and clinical implementation. Candidate predictor definitions and missing data handling were frequently only partially reported, and complete model specification was insufficient in most studies. In addition, only a minority of studies conducted external validation, and clinical utility evaluation was not consistently performed. Model presentation was commonly provided in the form of nomograms, but few studies supplied sufficient details, online calculators, or implementation tools to support independent reproduction or direct clinical use. These findings suggest that although basic model characteristics were generally reported, transparency regarding missing data handling, full model specification, external validation, and clinical usability remained limited across the included studies.

### 3.6. Core Predictors and Model Presentation Forms

Table 3 systematically summarizes the predictive factors and their presentation formats reported in the multivariable models of the included studies. Among the 34 prediction models from 32 included studies, the number of included predictors ranged from 3 to 12. These predictors could be broadly classified into five categories: demographic factors, disease-related factors, treatment-related factors, laboratory indicators, and other factors. To further synthesize recurring predictors across studies, the frequency of the most commonly reported predictor domains are summarized in Table S6. The top five predictor domains were age, operation/anesthesia time, preoperative nutritional indicators, preoperative inflammatory indicators, and American Society of Anesthesiologists (ASA) physical status classification.

Among these predictors, age was the most commonly incorporated demographic factor, reflecting the close association between older age and vulnerability to POD. Operation/anesthesia time represented treatment-related exposure and was frequently used to reflect surgical complexity and perioperative physiological burden. Preoperative nutritional indicators, such as albumin, prognostic nutritional index, Controlling Nutritional Status, Geriatric Nutritional Risk Index, and albumin-to-fibrinogen ratio, reflected baseline nutritional reserve. Preoperative inflammatory indicators, including neutrophil-to-lymphocyte ratio, systemic immune-inflammation index, C-reactive protein, and interleukin-related markers, reflected systemic inflammatory status. ASA physical status classification was also commonly included as an overall indicator of preoperative physical condition and perioperative risk.

Regarding model presentation, 28 studies used visual formats to display the models, with a visualization rate of 87.50%. Among them, the nomogram was the most commonly used form, which was applied in 22 studies.

## 4. Discussion

This scoping review systematically maps the current landscape of prediction models for POD in cancer patients. Over 90% of the relevant studies were published within the past five years, indicating rapid growth in this research area. However, the included studies showed substantial heterogeneity in delirium incidence estimation, assessment instrument selection, predictive factor identification, and modeling approaches, with the vast majority of evidence originating from Chinese populations, suggesting a pronounced geographical imbalance. Whereas prior reviews have largely focused on estimating incidence and identifying associated risk factors [4,48], our study shifts the analytical focus from individual risk factors to the prediction model as a whole. Adopting a scoping review methodology, this study systematically examines existing models across four dimensions: study design, modeling methods, model performance evaluation, and adherence to reporting standards. The findings reveal systematic deficiencies in model construction paradigms, validation strategies, and evaluation criteria. Rather than offering a single definitive conclusion, this review provides a structured reference framework to guide the standardized design of future modeling studies and to inform cross-population validation efforts.

### 4.1. Substantial Heterogeneity in the Incidence of POD Among Cancer Patients

The incidence of POD in cancer patients ranged from 6.7% to 46.39%, demonstrating substantial heterogeneity. This variation may be attributable to multiple factors. First, the composition of cancer types varied considerably across the included studies. Surgical procedures associated with different cancers may also directly affect POD risk. Among specific cancer types, esophageal cancer showed the highest POD incidence, ranging from 16.99% to 46.39%, which is consistent with previous findings [49]. Esophageal cancer surgery often requires one-lung ventilation and is associated with intraoperative blood pressure fluctuations and restrictive fluid management. These factors may lead to cerebral hypoperfusion and an imbalance in cerebral oxygen supply and demand [50]. In addition, extensive surgical trauma and a pronounced systemic inflammatory response may contribute to the high POD incidence in this population.

Gastric cancer and colorectal cancer also showed relatively high POD incidence rates, ranging from 14.50% to 36.43% and from 13.00% to 21.60%, respectively. This elevation may be associated with disruption of gut microbiota composition and intestinal barrier integrity after major abdominal surgery. These changes may increase systemic translocation of microbial products and subsequently induce postoperative neuroinflammation [51]. Furthermore, existing studies have demonstrated an elevated POD risk among patients undergoing head and neck surgery [48,52,53]. Consistently, the reported incidence range of POD following head and neck surgery in the present review ranged from 13.28% to 26.00%. In contrast, lung cancer surgery is associated with a relatively lower POD incidence. Zhu et al. reported a POD rate of only 6.7% in this patient group [9]. This may be attributed to the predominant use of minimally invasive surgery and the patients’ relatively good overall physical status, with a mean ASA score of 2.0. These findings indicate that cancer type and surgical procedure may substantially influence the heterogeneity of POD risk, suggesting that future POD prediction models should be developed and validated according to cancer type and surgical procedure to enhance clinical applicability.

Second, the selection of delirium assessment instruments varied considerably across the 32 included studies. Delirium is clinically categorized into hyperactive, hypoactive, and mixed subtypes [21]. Some assessment tools can also capture subsyndromal delirium [54], but their sensitivity for detecting different subtypes differs notably. Specifically, the DSM-5 not only delineates diagnostic criteria for delirium but also provides specific guidance on differential diagnosis, subtype classification, severity assessment, and etiological analysis [55]. The 3D-CAM has demonstrated favorable diagnostic accuracy for delirium detection across diverse care settings [56]. It was originally derived and validated as a brief diagnostic interview for CAM-defined delirium in older hospitalized medical patients [57]. In contrast, the CAM-ICU is predominantly used in intensive care unit settings. Its initial step involves assessing sedation status using the RASS score, which may facilitate subtype classification in ICU patients. However, its utility for delirium subtype assessment in non-ICU patients is relatively limited [58]. Accordingly, to enhance assessment accuracy, combining the CAM-ICU with other instruments such as the DSM-5 may improve assessment accuracy in clinical practice [59].

Furthermore, the timing of delirium assessment also affects incidence estimation: variations in the duration of the assessment window influence the number of delirium episodes captured, contributing to the observed differences in reported POD rates across studies. Therefore, future research should adopt assessment instruments tailored to specific ward settings and standardize screening time points to improve the accuracy of incidence estimates and enhance comparability across studies.

### 4.2. Insufficient Inclusion of Existing Predictive Factors

Our findings indicate that the five most common predictors were age, duration of surgery or anesthesia, preoperative nutritional indicators, preoperative inflammatory indicators, and ASA physical status classification. However, high frequency does not equate to comprehensiveness. The frequency ranking merely reflects the focus of existing research and does not imply that these factors constitute a complete predictive framework. On the contrary, several variables that have been confirmed to be closely associated with POD remain inadequately covered in current models.

Preoperative cognitive impairment has been shown to predict the occurrence of POD in cancer patients [48]. Its assessment directly reflects the reserve capacity of the central nervous system and its vulnerability to perioperative stress. Nevertheless, the vast majority of included studies did not incorporate it into their model analyses. A similar situation was observed for frailty status. Cancer and frailty are closely interrelated, with over half of older cancer patients exhibiting frailty [60]. When cancer coexists with frailty, stressors such as surgery and chemotherapy may further increase the risk of POD [61,62]. Previous studies have confirmed that frailty is an independent risk factor for POD in older cancer patients, increasing the risk of complications and delirium by approximately 2–3 fold [63]. Yet, only 3 studies included this variable in their analysis of potential predictors.

Anxiety and depression also merit attention. Anxiety and depressive symptoms are highly prevalent among cancer patients. They may affect quality of life and treatment adherence and have also been associated with POD and adverse surgical outcomes [64,65]. However, most included studies did not incorporate these variables into their analyses. The absence of these variables in most studies may limit the ability of current models to capture the multifactorial mechanisms of POD. It may also weaken their capacity to identify specific high-risk subgroups. Therefore, future studies should systematically synthesize candidate predictors from existing research. Variables with both clinical accessibility and predictive value should be incorporated through a combination of statistical screening and expert consultation. Such an integrated approach would enhance model coverage and predictive performance.

### 4.3. Methodological Limitations and High Risk of Bias in Model Development and Validation

Most included studies (n = 31) were cohort studies, which are generally more suitable than cross-sectional designs for establishing the temporal sequence between candidate predictors and POD occurrence [66]. However, one included study adopted a cross-sectional design, which limited the ability to confirm whether the candidate predictors preceded the outcome. Therefore, its findings should be interpreted cautiously and should mainly be considered as part of the evidence map rather than as strong evidence for clinical prediction.

More than half of the included studies used a retrospective design. Although retrospective cohorts are feasible and commonly used in prediction model research, they depend heavily on the completeness and accuracy of medical records. In the context of POD, retrospective identification based on descriptive nursing notes or routine clinical documentation, rather than standardized bedside delirium assessment, may lead to outcome misclassification. Inconsistent documentation of perioperative variables may also affect the reliability of candidate predictors. These limitations may introduce selection bias, information bias, and residual confounding, thereby weakening the reliability and applicability of the derived POD prediction models.

At the model development level, the high risk of bias was mainly driven by limitations in the analysis domain of the PROBAST assessment. Many studies had relatively small sample sizes, and several models failed to meet the commonly recommended threshold of at least 10 events per predictor variable. Insufficient events relative to the number of candidate predictors can produce unstable regression coefficients, increase the likelihood of overfitting, and lead to overly optimistic estimates of model performance. Recent methodological guidance emphasizes that prediction model development should be supported by adequate sample size planning, appropriate handling of missing data, prespecified candidate predictors, and internal validation to quantify and correct optimism [67,68]. In addition, some studies relied on univariable analysis for predictor screening. This approach may exclude clinically important predictors that do not reach statistical significance in univariable analysis and may also favor data-driven variable selection, reducing the reproducibility of the final model. Furthermore, incomplete reporting or inadequate handling of missing data may compromise sample representativeness and introduce additional bias.

Model validation and performance assessment were also insufficient in several studies. All included studies reported discrimination metrics, predominantly AUC or C-index, and 71.88% of the studies reported discrimination values above 0.80. However, good discrimination in the development dataset alone does not guarantee reliable clinical prediction. Calibration, which reflects the agreement between predicted and observed risks, is essential for determining whether a model can provide accurate individual risk estimates for clinical decision-making [67]. Without calibration assessment, a model with apparently good discrimination may still overestimate or underestimate the absolute risk of POD in specific patient groups. Moreover, 43.75% of the studies only evaluated model performance in the development dataset, only 25.00% conducted external validation, and 43.75% did not assess clinical utility. External validation is necessary to examine whether a model can maintain adequate discrimination, calibration, and clinical usefulness in independent populations or different clinical settings [69]. Although internal validation can estimate optimism within the original dataset, it cannot determine model transportability across different clinical environments. This issue is particularly relevant for POD prediction, as POD risk may be influenced by cancer type, surgical complexity, anesthesia strategy, perioperative care pathway, postoperative monitoring, and delirium assessment practice. Therefore, models developed in single-center or highly selected cohorts may not perform equally well in other institutions or healthcare systems. Future studies should prioritize external validation of existing models in multicenter prospective cohorts before developing additional new models.

Machine-learning methods, including random forest and XGBoost, have been increasingly applied in recent years. Compared with traditional regression models, machine-learning algorithms may capture nonlinear relationships and complex interactions among predictors [70]. However, their advantages depend on adequate sample size, appropriate data partitioning, transparent reporting of model tuning, and rigorous validation. Recent TRIPOD + AI guidance emphasizes transparent reporting for prediction models using regression or machine-learning methods, including data preprocessing, predictor handling, model specification, hyperparameter tuning, validation, and reproducibility [71]. In the included studies, machine-learning models were not consistently accompanied by sufficient external validation, calibration assessment, or clinical utility evaluation. Therefore, although some machine-learning models showed promising discrimination, their added value over conventional regression models remains uncertain without robust validation and transparent reporting [72].

Overall, the methodological limitations identified in existing studies may directly affect the clinical applicability of POD prediction models in cancer patients. Small sample sizes, data-driven predictor selection, poor handling of missing data, inadequate correction for overfitting, incomplete calibration assessment, and insufficient external validation can reduce model stability, reproducibility, and transportability. Future studies should prioritize prospective multicenter designs, standardized POD assessment, adequate sample size planning, appropriate missing data methods, internal validation with optimism correction, external validation in independent cohorts, and evaluation of both calibration and clinical utility before recommending these models for routine perioperative oncology practice.

### 4.4. Deficiencies in Reporting Quality, Model Presentation, and Clinical Usability

Transparent reporting is essential for the reproduction, external validation, and clinical implementation of prediction models. In this review, although most studies clearly reported POD assessment methods, assessment windows, POD event numbers, and predictor selection strategies, several TRIPOD-relevant items were incompletely reported. In particular, candidate predictor definitions, missing data handling, and complete model specification were frequently insufficient. Many studies presented models as nomograms or scoring systems, but did not provide full regression equations, intercepts, coding rules, online calculators, or implementation tools. This limits the ability of independent researchers to reproduce the models and evaluate their performance in new clinical settings. In addition, external validation and clinical utility evaluation were not consistently performed, which further restricts the translation of these models into routine perioperative oncology practice. Future studies should improve transparent reporting by providing complete model specifications, standardized predictor definitions, detailed missing data methods, calibration results, decision-curve analysis, and accessible tools when appropriate.

Regarding model presentation, 87.50% of the included studies used visual formats to display the POD prediction models, with the nomogram being the most commonly used form. This indicates a relatively high level of awareness of model visualization in current research. Nomograms have gained wide acceptance because of their intuitive design and ease of bedside use. It should be noted, however, that the interpretability provided by nomograms is primarily applicable to traditional statistical models such as logistic regression. A nomogram essentially converts regression coefficients into a visual scoring tool. This allows clinicians to directly observe the risk assignment of each variable, but it does not reveal interactions among variables or capture nonlinear effects [73]. In contrast, among the increasing number of machine-learning models, only 1 study employed Local Interpretable Model-agnostic Explanation (LIME) for post hoc interpretability analysis. The remaining 3 studies only reported feature importance plots or waterfall plots. They did not provide more detailed interpretability data, such as Shapley Additive Explanations (SHAP) values, partial dependence plots, or force plots. This implies that although machine-learning algorithms such as random forest may outperform traditional regression in terms of discrimination, their internal decision-making processes remain a black box to clinical users, making it difficult to gain sufficient trust for bedside decision-making [74].

Different modeling approaches involve a trade-off between predictive performance and clinical interpretability. Simpler regression-based models are easier to understand, reproduce, and implement at the bedside, but may have limited ability to capture nonlinear effects or complex predictor interactions. In contrast, more complex machine-learning models may improve discrimination, but they require larger datasets, careful feature selection, parameter tuning, and external validation to reduce overfitting and support generalizability. Given these considerations, future studies should evaluate not only AUC or C-index, but also calibration, clinical utility, interpretability, and implementation feasibility. Furthermore, future studies should adopt interpretability strategies aligned with the modeling approach employed: traditional models may continue to rely on nomograms for visualization, whereas machine-learning models should include SHAP or LIME analyses as a standard component of model reporting, in order to bridge the gap between algorithmic performance and clinical credibility.

### 4.5. Limitations

This review has several limitations that should be considered. First, only published studies in English and Chinese were included, while studies published in other languages were not searched, which may have led to the omission of relevant evidence. In addition, most included studies were conducted in China, which may limit the generalizability of the findings to other healthcare systems, populations, and perioperative oncology practices. Differences in patient characteristics, cancer types, anesthesia strategies, postoperative care pathways, delirium assessment practices, and nursing workflows may influence both POD incidence and model performance. Second, the included studies exhibited substantial heterogeneity in POD assessment instruments, screening time points, and modeling methods, which precluded meta-analysis. Accordingly, this review was limited to a systematic synthesis of the available evidence without pooled quantitative effect estimates. Another limitation is the potential cohort overlap or data reuse among some included studies. Although we assessed possible overlap using available study information, such as authorship, institution, study period, sample size, cancer type, surgical procedure, registration number, and data source, overlap could not be completely ruled out. Therefore, these studies were flagged and interpreted cautiously rather than regarded as fully independent evidence. One cross-sectional study was retained despite its high risk of bias because this scoping review aimed to map the breadth of available evidence in the field. This study provided supplementary information on the current status of POD risk factors and prediction model research, but its findings were interpreted cautiously and were not used to support causal inference or conclusions regarding model generalizability. Furthermore, several included studies reported model parameters incompletely, such as missing full regression coefficients or intercepts, which hindered deeper structural comparison across models and reflected ongoing deficiencies in reporting transparency.

Future research should prioritize multicenter prospective validation of existing models across different populations, institutions, and healthcare systems to evaluate their generalizability, robustness, and clinical transportability. Standardized delirium assessment methods should also be adopted, including the use of validated tools (such as CAM, 3D-CAM, or CAM-ICU), clearly defined assessment windows, repeated postoperative screening, and trained assessors, so as to reduce outcome misclassification and improve comparability across studies. Model development should also be standardized at both the data and modeling levels. This includes establishing a core predictor set, standardizing data collection procedures, reporting complete model specifications, and strengthening both internal and external validation. Greater attention should be paid to underrepresented but clinically relevant predictors, such as preoperative cognitive function, frailty, anxiety, and depression. When feasible, neurophysiological monitoring indicators such as electroencephalogram signals [75] may also be incorporated to enrich multidimensional data sources. Furthermore, machine-learning models should be reported transparently, including data preprocessing, missing data handling, class imbalance handling, feature selection, hyperparameter tuning, model calibration, code or tool availability where possible, and interpretability analyses such as SHAP or LIME. Future studies should also provide decision-curve analysis, accessible online calculators or implementation tools, and prospective impact evaluation to determine whether these models can improve clinical decision-making and patient outcomes. These steps may support the transition of POD prediction models from model development to safe clinical application.

## 5. Conclusions

Over the past five years, the application of prediction models for POD in cancer patients has grown substantially, reflecting sustained academic interest in this area. This review systematically mapped the current landscape of POD prediction models in cancer patients. The main findings are as follows. Existing models are predominantly based on traditional logistic regression. Although machine-learning algorithms have been increasingly explored, their use remains limited. The overall discrimination of the models is acceptable. However, external validation and clinical utility assessment are generally insufficient, and most studies remain at the model development stage. Key predictors such as preoperative cognitive function, frailty status, and emotional disturbance are insufficiently covered. In addition, the interpretability of machine-learning models requires further strengthening. Overall, this field is transitioning from model development to clinical application. Future research should focus on standardized methodology, multicenter external validation, transparent reporting, and prospective evaluation of clinical impact. Researchers with an interest in predictive analysis and cancer POD prediction models should adopt the TRIPOD and PROBAST standards early in model planning to improve model quality, reproducibility, and clinical applicability.

## Figures and Tables

**Table 1:** Basic characteristics of included studies (n = 32).

Authors/Year/ Country	Study Population	Data Collection	Sample Size	Study Type	POD Incidence	Modeling Method	Discriminant Validity	Calibration	Clinical Benefit	Validation Method	Average Age (Years)
Choi et al. (2017) [11], South Korea	Head and neck cancer	Retrospective	341	Cohort study	26.00%	①	D: 0.7407 a V: 0.6898 a	/	/	Cross-validation	56 ± 12
Flanigan et al. (2018) [13], USA	Glioblastoma	Retrospective	554	Cohort study	7.00%	①	D: 0.82 b V: 0.82 b	D: 0.07 d V: 0.07 d	/	External validation	60.8 ± 12.8
Mosk et al. (2018) [42], the Netherlands	Colorectal cancer	Retrospective	251	Cohort study	13.00%	①	LSMM + malnourishment: 0.795 b LSMM + physical dependency: 0.783 b	LSMM + malnourishment: 0.096 d LSMM + physical dependency: 0.099 d	/	/	76 (73, 80)
Yajima et al. (2023) [14], Japan	Urological cancer	Retrospective	541	Cohort study	D: 7.00% V: 14.00%	①	D: 0.819 a V: 0.804 a	⑰	/	External validation	D: 72 (68, 77) V: 71 (65, 75)
Shen and Xu (2024) [24], China	Esophageal cancer	Prospective	194	Cohort study	46.39%	①②	②: 0.837 a	/	/	/	PG: 61.84 ± 8.62 NG: 57.10 ± 7.81
Shen and Wang (2025) [25], China	Oral cancer	Prospective	106	Cohort study	12.26%	①	D: 0.785 a V: 0.762 a	D: 0.814 c V: 0.748 c	/	Random split	/
Shen et al. (2023) [26], China	Head and neck cancer	Prospective	128	Cohort study	13.28%	①	0.913 a	/	/	/	61.14 ± 10.06
Chen et al. (2026) [27], China	Lung cancer	Retrospective	597	Cohort study	23.50%	①	D: 0.804 a V: 0.793 a	D: 0.231 c V: 0.373 c	DCA	Random split	D: 77.00 (71.75, 81.00) V: 78.00 (73.00, 82.00)
Chen et al. (2023) [28], China	Gastric cancer	Prospective	541	Cohort study	14.50%	①	D: 0.766 a V: 0.770 a	D: 0.576 c V: 0.490 c	DCA	Bootstrap method, external validation	/
Chu et al. (2025) [29], China	Oral cancer	Retrospective	255	Cohort study	14.90%	①	0.917 a/0.834 b	⑰	DCA	Bootstrap method	PG: 79.37 ± 7.85 NG: 69.19 ± 5.70
Gao and Huan (2025) [30], China	Gastric cancer	Prospective	390	Cohort study	20.51%	①	D: 0.854 a V: 0.812 a	/	DCA	Random split, Bootstrap method	PG: 73.45 ± 5.23 NG: 73.31 ± 5.20
Li et al. (2022) [31], China	Lung cancer	Retrospective	580	Cohort study	7.93%	①	0.866 a/0.864 b	⑰	DCA	Bootstrap method	/
Liang and Xie (2023) [32], China	Prostate cancer	Prospective	657	Cohort study	16.59%	①	0.895 b	⑰	DCA	Bootstrap method	/
Tang et al. (2025) [33], China	Colorectal cancer	Retrospective	515	Cohort study	13.20%	①	D: 0.902 a V: 0.933 a	⑰	DCA	External validation	PG (D): 68.8 ± 5.4 NG (D): 67.3 ± 4.4 PG (V): 69.4 ± 3.9 NG (V): 67.1 ± 4.7
Weng et al. (2024) [34], China	Urological oncology	Prospective	1180	Cohort study	9.40%	①③④⑤⑥⑦	D③: 0.889 a V③: 0.882 a	/	/	Random split	/
Xu et al. (2025) [35], China	Colorectal cancer	Retrospective	450	Cohort study	17.00%	①	D: 0.889 a/0.883 b V: 0.931 a/0.928 b	D: 0.877 c/⑰ V: 0.988 c/⑰	/	External validation	/
Ye et al. (2022) [36], China	Gastrointestinal tumor	Prospective	583	Cross-sectional study	16.30%	①	0.784 a	0.090 c	/	/	60.44 ± 12.21
Zhang et al. (2023) [37], China	Laryngeal cancer	Prospective	561	Cohort study	16.20%	①	D: 0.803 a V: 0.790 a	D: 0.499 c/⑰ V: 0.573 c/⑰	/	External validation	D: 67.6 ± 4.9 V: 67.4 ± 5.3
Zhang et al. (2025) [38], China	Lung cancer	Prospective	618	Cohort study	19.26%	①	D: 0.856 a V: 0.832 a	/	/	Random split	/
Chen et al. (2025a) [39], China	Esophageal cancer	Retrospective	924	Cohort study	16.99%	①	0.832 b	⑰	DCA	/	PG: 69.73 ± 7.36 NG: 66.12 ± 6.84
Chen et al. (2025b) [12], China	Oral cancer	Retrospective	359	Cohort study	26.50%	①	D: 0.82 a V: 0.84 a	D: 0.63 c/⑰ V: 0.57 c/⑰	DCA	Random split, Bootstrap method	68.00 (65.00, 72.00)
Hu et al. (2024) [40], China	Abdominal malignant tumor	Retrospective	611	Cohort study	22.91%	①	P1: 0.862 a P2: 0.856 a	P1: 0.784 c P2: 0.990 c	/	/	PG: 71.00 ± 3.32 NG: 70.37 ± 4.45
Liu et al. (2023) [41], China	Gastric cancer	Retrospective	280	Cohort study	36.43%	①	0.903 a	/	/	/	63.48 ± 5.29
Shen et al. (2025) [43], China	Esophageal cancer	Retrospective	396	Cohort study	35.40%	①	D: 0.919 a/0.92 b V: 0.871 a/0.87 b	D: 0.939 c/0.106 d/⑰ V: 0.456 c/0.115 d/⑰	DCA	Random split, Bootstrap method	68.7 ± 6.4
Wan et al. (2025) [10], China	Colorectal cancer	Prospective	555	Cohort study	18.02%	①③④⑤⑧⑨⑩⑪⑫⑬	③: 0.795 a	/	DCA	Random split, Cross-validation	65 (56, 71)
Wang et al. (2025) [44], China	Prostate cancer	Prospective	156	Cohort study	15.38%	①	0.973 a/0.963 b	⑰	DCA	Cross-validation	/
Xiang et al. (2023) [16], China	Gynecologic cancer	Retrospective	226	Cohort study	17.30%	①	0.833 a	⑰	DCA	/	70.6
Xue et al. (2025) [17], China	Lung cancer	Retrospective	1066	Cohort study	19.04%	①	D: 0.871 a V: 0.914 a	D: 0.29 c/⑰ V: 0.72 c/⑰	/	External validation	/
Yan et al. (2024) [45], China	Hepatocellular carcinoma	Retrospective	1481	Cohort study	13.30%	①	D: 0.798 a V: 0.808 a	⑰	DCA	External validation	D: 70.1 ± 4.9 V: 69.8 ± 4.6
Zhao et al. (2024) [46], China	Colorectal cancer	Prospective	334	Cohort study	21.60%	①	0.841 a	0.536 c	/	/	65 (56, 71)
Zhu et al. (2026) [9], China	Lung cancer	Retrospective	2570	Cohort study	6.70%	①②③④⑤⑧⑪⑫⑬⑭⑮⑯	④: 0.763 a	/	/	Cross-validation	69.6 ± 9.0
Zhu et al. (2025) [15], China	Cervical cancer	Retrospective	253	Cohort study	16.20%	①	D: 0.821 a V: 0.966 a	D: 0.395 c/⑰ V: 0.319 c/⑰	DCA	External validation	PG: 72.3 ± 3.7 NG: 70.4 ± 3.7

**Abbreviations:** decision curve analysis (DCA); POD group (PG); non-POD group (NG); development cohort (D); validation cohort (V); original predictive model (P1); synthetic minority oversampling technique (SMOTE)-based logistic early warning model (P2); low skeletal muscle mass (LSMM). a denotes the AUROC value; b refers to the C-index; c represents the *p*-value of the Hosmer–Lemeshow test; d refers to Brier score. **Note:** ① logistic regression model; ② decision tree; ③ random forest; ④ support vector machines (SVMs); ⑤ eXtreme Gradient Boosting (XGBoost); ⑥ artificial neural network (ANN); ⑦ Bayesian network (BN); ⑧ k-nearest neighbor algorithm (KNN); ⑨ neural network (NN); ⑩ gradient boosting machine (GBM); ⑪ adaptive boosting (AdaBoost); ⑫ categorical boosting (CatBoost); ⑬ light gradient boosting machine (LightGBM); ⑭ multilayer perceptron (MLP); ⑮ Gaussian Naive Bayes; ⑯ gradient boosting decision trees (GBDT); ⑰ calibration curve.

**Table 2:** Risk of bias and applicability assessment of included study using the PROBAST (n = 32).

Authors/Year/Country	Participants	Predictors	Outcome	Analysis	Applicability	Overall
Choi et al. (2017) [11], South Korea	Low	High	Low	High	High	High
Flanigan et al. (2018) [13], USA	Low	Low	Low	High	Low	High
Mosk et al. (2018) [42], The Netherlands	Low	Low	Low	High	Low	High
Yajima et al. (2023) [14], Japan	Low	Low	Low	Low	Low	Low
Shen and Xu (2024) [24], China	Low	High	Low	High	High	High
Shen and Wang (2025) [25], China	Low	High	Low	High	High	High
Shen et al. (2023) [26], China	Low	Low	Low	High	Low	High
Chen et al. (2026) [27], China	Low	Low	Low	Low	Low	Low
Chen et al. (2023) [28], China	Low	Low	Low	Low	Low	Low
Chu et al. (2025) [29], China	Low	Low	Low	High	Low	High
Gao and Huan (2025) [30], China	Low	High	Low	High	High	High
Li et al. (2022) [31], China	Low	High	Unclear	High	High	High
Liang and Xie (2023) [32], China	Low	High	Unclear	High	High	High
Tang et al. (2025) [33], China	Low	Low	Low	High	Low	High
Weng et al. (2024) [34], China	Low	Low	Low	High	Low	High
Xu et al. (2025) [35], China	Low	Low	Low	Low	Low	Low
Ye et al. (2022) [36], China	Low	High	High	High	High	High
Zhang et al. (2023) [37], China	Low	Unclear	Low	High	Unclear	High
Zhang et al. (2025) [38], China	Low	High	Low	High	High	High
Chen et al. (2025a) [39], China	Low	High	Low	High	High	High
Chen et al. (2025b) [12], China	Low	High	Low	High	High	High
Hu et al. (2024) [40], China	Low	High	Low	High	High	High
Liu et al. (2023) [41], China	Low	High	Low	High	High	High
Shen et al. (2025) [43], China	Low	High	Low	Low	High	High
Wan et al. (2025) [10], China	Low	Low	Low	Low	Low	Low
Wang et al. (2025) [44], China	Low	Low	Low	High	Low	High
Xiang et al. (2023) [16], China	Low	Low	Low	High	Low	High
Xue et al. (2025) [17], China	Low	High	Low	Low	High	High
Yan et al. (2024) [45], China	Low	Low	Low	Low	Low	Low
Zhao et al. (2024) [46], China	Low	High	Low	High	High	High
Zhu et al. (2026) [9], China	Low	Low	Low	High	Low	High
Zhu et al. (2025) [15], China	Low	Low	Low	High	Low	High

**Note:** PROBAST: Prediction model Risk Of Bias ASsessment Tool. Low risk: good study design and methodological quality. High risk: major flaws that may affect model performance or generalizability. Unclear risk: insufficient information to make a judgment. The evaluation criteria for “Low” and “High” risk were based on the PROBAST [23].

**Table 3:** Model predictive factors and presentation forms (n = 32).

Authors/Year/Country	Predictive Factors (OR/β, 95% CI)	Presentation Format
Choi et al. (2017) [11], South Korea	Age (1.03, 1.00–1.05), history of psychiatric disorder (11.7, 1.14–120.33), married marital status (0.01, 0.002–0.10), ex-married marital status (0.07, 0.01–0.30), preoperative NRS (1.20, 1.07–1.35), ASA classification (1.77, 1.08–2.89), ICU stay period (1.01, 1.00–1.02)	②
Flanigan et al. (2018) [13], USA	Age (2.7, 1.5–5.0), chronic pulmonary disease (3.9, 1.3–12.0), psychiatric history (5.9, 2.5–14.0), bihemispheric tumors (2.5, 1.1–5.8), tumor size (2.8, 1.5–5.3)	④
Mosk et al. (2018) [42], The Netherlands	LSMM + malnourishment: Age (1.12, 1.04–1.20), history of delirium (6.70, 2.20–20.42), LSMM combined with malnourishment (4.00, 1.25–12.82) LSMM + physical dependency: Age (1.11, 1.04–1.20), history of delirium (5.28, 1.72–16.20), LSMM combined with physical dependency (4.32, 1.30–14.36)	②
Yajima et al. (2023) [14], Japan	Mini-Cog score < 3 (9.5, 4.2–21.4), disability in the responsibility for medication (4.1, 1.1–14.7), preoperative use of benzodiazepine (6.4, 2.6–15.7)	None
Shen and Xu (2024) [24], China	Age (1.075, 1.029–1.123), excessive alcohol consumption history (2.228, 1.087–4.568), deep sedation (2.784, 1.360–5.699), intraoperative hypotension (2.587, 1.278–5.237), postoperative pain score (1.971, 1.397–2.782), hemoglobin (0.964, 0.945–0.984)	①
Shen and Wang (2025) [25], China	Age ≥ 60 years (6.585, 1.252–29.858), decreased PNI (6.126, 1.012–22.639), elevated SII (6.492, 1.238–25.128), blood transfusion (0.317, 3.609–77.551), sleep disturbance (3.330, 2.514–58.257), high VAS score (0.937, 0.843–21.997)	None
Shen et al. (2023) [26], China	Age ≥ 65 years (5.253, 1.146–24.074), preoperative NLR (1.891, 1.050–3.405), intraoperative blood transfusion (6.108, 1.109–33.644), postoperative sleep disorder (9.292, 1.441–59.914), postoperative pain (1.807, 1.018–3.206)	None
Chen et al. (2026) [27], China	Age (1.30, 1.070–1.193), education level (0.581, 0.344–0.982), preoperative cognitive function (0.821, 0.745–0.904), history of cerebrovascular disease (2.667, 1.325–5.367), operative time (1.023, 1.010–1.036)	②
Chen et al. (2023) [28], China	Age (2.803, 1.597–4.919), preoperative nutrition risk (1.785, 1.034–3.081), operation mode (1.914, 1.137–3.223), operation time (1.861, 1.061–3.263), intraoperative bleeding (1.970, 1.145–3.389)	②
Chu et al. (2025) [29], China	Age (1.194, 1.106–1.289), operation time (2.281, 1.476–3.527), postoperative electrolyte disturbance (8.603, 2.923–25.324)	②
Gao and Huan (2025) [30], China	Preoperative hypertension history (2.502, 1.223–5.381), respiratory rate (3.452, 1.723–6.925), creatinine value (2.012, 1.048–3.853), surgical methods (1.572, 0.726–3.394), operation time (1.821, 1.096–3.024), intraoperative blood loss (2.763, 1.423–5.364), mechanical ventilation (3.982, 1.987–7.972), APS III score (2.502, 1.277–4.894)	②
Li et al. (2022) [31], China	Aged ≥ 75 years (2.994, 1.394–6.431), preoperative MMSE score ≤ 25 points (8.320, 3.807–18.185), preoperative PNI < 45 (3.378, 1.244–9.176), CCI score ≥ 2 points (3.053, 1.065–8.748), squamous cell carcinoma (5.461, 1.383–21.565), intraoperative hypotension (20.643, 7.466–57.071), operating time ≥ 3 h (3.812, 1.403–10.355)	②
Liang and Xie (2023) [32], China	Age ≥ 75 years (7.605, 1.105–1.380), MMSE score < 27 points (6.501, 3.458–12.113), operation time ≥ 120 min (8.587, 4.814–19.390), intraoperative blood loss ≥ 600 mL (8.513, 4.734–19.446), SIINI ≥ 18.51 (8.605, 6.145–21.329)	②
Tang et al. (2025) [33], China	Advanced age (1.107, 1.015–1.208), prolonged operative time (1.019, 1.006–1.033), elevated NLR (25.939, 9.135–73.635), elevated mFI (9.097, 3.816–21.688), increased AFR (0.606, 0.414–0.888), increased PNI (0.896, 0.820–0.981)	②
Weng et al. (2024) [34], China	Age, diabetes, ASA grade, preoperative albumin, operation time	①
Xu et al. (2025) [35], China	Age (1.84, 1.57–3.10), intraoperative hypotension (1.40, 1.10–1.96), intraoperative hypoxia (1.64, 1.21–2.01), preoperative PNI (1.52, 1.02–1.87), preoperative NLR (1.81, 1.31–2.56), preoperative mFI (2.10, 1.77–2.65)	②
Ye et al. (2022) [36], China	Previous constipation (1.845, 1.106–3.078), use of mask oxygen inhalation (3.719, 1.322–10.459), transfer to ICU (3.258, 1.384–7.666), preoperative hypokalemia (0.402, 0.228–0.711), postoperative pain score (1.293, 1.139–1.467)	③
Zhang et al. (2023) [37], China	Age (2.172, 1.195–3.949), diabetes (2.511, 1.438–4.386), ASA classification (2.032, 1.138–3.629), preoperative serum albumin (2.201, 1.267–3.823)	②
Zhang et al. (2025) [38], China	Age > 79 years (2.221, 1.642–3.004), CCI score ≥ 2 points (1.540, 1.011–2.348), thoracotomy (1.902, 1.177–3.075), propofol dosage (1.413, 1.133–1.764)	②③
Chen et al. (2025a) [39], China	Age > 70 years (2.524, 1.698–3.750), use of penehyclidine hydrochloride (1.717, 1.132–2.604), open surgery (0.219, 0.075–0.639), preoperative lymphocyte ≤ 1.45 × 10^9^/L (2.113, 1.232–3.623), preoperative albumin ≤ 43.6 g/L (1.898, 1.109–3.249), preoperative PNI ≤ 50.9 (2.330, 1.245–4.358), preoperative NLR > 2.33 (2.081, 1.264–3.424), preoperative PWR ≤ 34.97 (2.012, 1.330–3.045), postoperative PNI ≤ 39.40 (2.078, 1.353–3.191)	②
Chen et al. (2025b) [12], China	Age (1.08, 1.01–1.15), male sex (2.33, 1.10–5.04), alcohol consumption history (3.16, 1.42–7.13), unmarried status (6.84, 1.26–39.41), widowed/divorced status (3.90, 1.41–10.87), preoperative anxiety (2.32, 1.16–4.67), preoperative sleep disorder (2.41, 1.20–5.02), ICU length of stay (1.65, 1.15–2.40)	②
Hu et al. (2024) [40], China	P1: CCI (2.113, 1.970–5.266), ASA classification (2.130, 1.586–4.691), history of cerebrovascular disease (3.261, 2.293–5.681), duration of surgery (3.235, 2.440–4.469), perioperative blood transfusion (2.895, 2.194–6.692), postoperative pain score (3.065, 2.610–4.725) P2: CCI (1.693, 1.494–5.388), ASA classification (1.723, 1.536–6.775), history of cerebrovascular disease (3.212, 1.930–3.397), duration of surgery (3.170, 2.326–7.894), perioperative blood transfusion (2.737, 1.247–3.682), postoperative pain score (3.101, 2.092–12.064)	③
Liu et al. (2023) [41], China	Age (1.598, 1.154–3.652), ASA classification (3.975, 1.654–5.021), anesthetic drug consumption (1.325, 1.035–2.564), extraction time (1.409, 1.098–3.246), PACU stay (1.168, 1.006–1.986), VAS scores after awakening (0.004, 0.002–0.013)	②
Shen et al. (2025) [43], China	Propofol use (9.78, 3.10–30.86), PNI (0.91, 0.83–0.98), duration of mechanical ventilation (1.22, 1.02–1.45), postoperative pain (10.08, 3.92–25.89), postoperative infection (3.17, 1.37–7.35), dexmedetomidine use (3.78, 1.59–8.99)	②
Wan et al. (2025) [10], China	Age, education level, hypertension, diabetes, CHD, anesthesia time, TG, TC, TMAO_T1, TMAO_T2, IL-1beta_T2, IL-6_T1	①②⑤
Wang et al. (2025) [44], China	Sleep disorders (12.931, 1.191–140.351), ACCI (2.608, 1.143–5.950), postoperative infection (19.298, 2.53–147.202), NRS (4.033, 1.062–15.324)	②
Xiang et al. (2023) [16], China	Age (2.01, 1.16–3.56), mFI ≥ 0.225 (1.82, 1.06–3.13), CRP ≥ 8.0 (1.67, 1.06–2.92), SII (2.07, 1.17–3.65), AFR (2.36, 1.36–3.89)	②
Xue et al. (2025) [17], China	Age (1.180, 1.143–1.219), BMI (0.834, 0.773–0.900), education level (0.886, 0.818–0.960), history of diabetes (1.335, 0.731–2.436), history of cerebrovascular disease (1.877, 0.844–4.175), surgical approach (thoracotomy) (1.319, 0.583–2.988), duration of surgery (1.017, 1.006–1.029), time to recovery from anesthesia (1.050, 1.009–1.093)	②③
Yan et al. (2024) [45], China	Age (1.853, 1.288–2.667), history of cerebrovascular disease (2.026, 1.223–3.355), ASA classification (2.044, 1.395–2.996), albumin level (1.479, 1.032–2.121), surgical approach (1.547, 1.064–2.249)	②
Zhao et al. (2024) [46], China	Education (0.345, 0.162–0.732), preoperative TC (1.376, 1.002–1.888), preoperative TG (1.264, 1.008–1.586), diet (0.914, 0.843–0.991), history of hypertension (2.091, 1.008–4.336), SAS < 4 (5.186, 2.232–12.050) and SAS > 4 (4.377, 1.669–11.481), postoperative TMAO (1.019, 1.042–1.181)	None
Zhu et al. (2026) [9], China	Preoperative blood glucose levels, VC, MCV, preoperative albumin levels	⑥
Zhu et al. (2025) [15], China	Advanced age (1.12, 1.01–1.24), depressed AFR (0.69, 0.49–0.96), elevated NLR (3.51, 1.71–7.21), CONUT score (1.81, 1.22–2.69), GNRI (0.94, 0.90–0.97)	②

**Abbreviations:** acute physiology score (APS), age-adjusted Charlson Comorbidity Index (ACCI), albumin-to-fibrinogen ratio (AFR), American Society of Anesthesiologists physical status classification (ASA), Charlson Comorbidity Index (CCI), Controlling Nutritional Status (CONUT), coronary heart disease (CHD), C-reactive protein (CRP), Geriatric Nutritional Risk Index (GNRI), intensive care unit (ICU), interleukin (IL), low skeletal muscle mass (LSMM), mean corpuscular volume (MCV), Mini-Mental State Examination (MMSE), modified frailty index (mFI), neutrophil-to-lymphocyte ratio (NLR), numerical rating scale (NRS), original predictive model (P1), platelet-to-white cell ratio (PWR), post-anesthesia care unit (PACU), postoperative time point (T2), preoperative time point (T1), prognostic nutritional index (PNI), Sedation-Agitation Scale (SAS), synthetic minority oversampling technique (SMOTE)-based logistic early warning model (P2), systemic immune-inflammation index (SII), systemic immune-inflammatory-nutritional index (SIINI), total cholesterol (TC), triglyceride (TG), trimethylamine N-oxide (TMAO), vital capacity (VC), visual analog scale (VAS). **Note:** ① Shapley Additive Explanations (SHAP) plot; ② nomogram; ③ prediction model formula derived from regression coefficients of factors; ④ scoring system; ⑤ online nomogram prediction tool using Shiny; ⑥ visualized using Local Interpretable Model-agnostic Explanation (LIME) tool.

## Data Availability

The original contributions presented in this study are included in the article and Supplementary Materials. Further inquiries can be directed to the corresponding authors.

## Supplementary Materials

The following supporting information can be downloaded at: https://www.mdpi.com/article/10.3390/healthcare14142207/s1, Table S1. Database search terms and results. Table S2. Assessment of potential cohort overlap or data reuse among included studies. Table S3. Diagnostic criteria for POD in included studies (n = 32). Table S4. Comparative summary of machine-learning-based POD prediction models. Table S5. Key reporting characteristics of included prediction model studies according to selected TRIPOD-relevant items (n = 32). Table S6. Frequency of the most commonly reported predictor domains across included studies.

## Abbreviations

The following abbreviations are used in this manuscript:

ACCI	age-adjusted Charlson Comorbidity Index
AFR	albumin-fibrinogen ratio
APS	acute physiology score
ASA	American Society of Anesthesiologists physical status classification
3D-CAM	3-Minute Diagnostic Interview for CAM-defined Delirium
4AT	Four-item Acute Delirium Test
CAM	Confusion Assessment Method
CAM-ICU	Confusion Assessment Method for the Intensive Care Unit
CCI	Charlson Comorbidity Index
CHD	coronary heart disease
CONUT	Controlling Nutritional Status
CRP	C-reactive protein
DCA	decision curve analysis
DSM	Diagnostic and Statistical Manual of Mental Disorders
GNRI	Geriatric Nutritional Risk Index
ICU	intensive care unit
IL	interleukin
LIME	Local Interpretable Model-agnostic Explanation
LR	logistic regression
LSMM	low skeletal muscle mass
MCV	mean corpuscular volume
mFI	modified frailty index
MMSE	Mini-Mental State Examination
NLR	neutrophil-to-lymphocyte ratio
NRS	numerical rating scale
PACU	post-anesthesia care unit
PNI	prognostic nutritional index
POD	postoperative delirium
PROBAST	Prediction model Risk Of Bias ASsessment Tool
PWR	platelet-to-white cell ratio
RF	random forest
SAS	sedation-agitation scale
SHAP	Shapley Additive Explanations
SII	systemic immune-inflammation index
SIINI	systemic immune-inflammatory-nutritional index
SVM	support vector machine
TC	total cholesterol
TG	triglyceride
TMAO	trimethylamine N-oxide
TRIPOD	Transparent Reporting of a multivariable prediction model for Individual Prognosis Or Diagnosis
VAS	visual analog scale
VC	vital capacity
XGBoost	extreme gradient boosting
