# Supplementary material for: Prediction Models for Postoperative Delirium Among Cancer Patients: A Scoping Review
Source: Healthcare (Basel). 2026 Jul 21;14(14):2207. doi: 10.3390/healthcare14142207 (PMC13411249; doi:10.3390/healthcare14142207)
Supplement: Supplementary file 1 [file healthcare-14-02207-s001.zip › healthcare-4379671-supplementary.pdf]

Supplementary file lists

Table S1. Database search terms and results.

Table S2. Assessment of potential cohort overlap or data reuse among included studies.

Table S3. Diagnostic criteria for POD in included studies (n=32).

Table S4. Comparative summary of machine-learning-based POD prediction models.

Table S5. Key reporting characteristics of included prediction model studies according to selected TRIPOD-relevant items (n=32).

Table S6. Frequency of the most commonly reported predictor domains across included studies.

Table S1. Database search terms and results.

Database	Search strategy	Search results (*n*)
CINAHL	((MH "Neoplasms+" OR MH "Carcinoma+" OR TI(neoplasms OR carcinoma OR cancer OR cancer* OR oncology OR carcinoma* OR tumor* OR tumours OR tumour OR neoplasm OR neoplasm* OR malignan* OR metasta* OR sarcoma* OR glioma*) OR AB(neoplasms OR carcinoma OR cancer OR cancer* OR oncology OR carcinoma* OR tumor* OR tumours OR tumour OR neoplasm OR neoplasm* OR malignan* OR metasta* OR sarcoma* OR glioma*))) AND ((MH "Delirium+" OR TI(delirium OR delirium* OR deliri* OR "postoperative delirium" OR "post operative delirium" OR "postsurgical delirium" OR "postoperative cognitive dysfunction" OR "emergence delirium" OR "acute confusion*" OR "temporary confusion*" OR "subacute delirium*") OR AB(delirium OR delirium* OR deliri* OR "postoperative delirium" OR "post operative delirium" OR "postsurgical delirium" OR "postoperative cognitive dysfunction" OR "emergence delirium" OR "acute confusion*" OR "temporary confusion*" OR "subacute delirium*"))) AND ((TI("risk prediction" OR "risk score" OR "risk model" OR "prognostic model" OR "prediction model" OR "risk assessment" OR "risk stratification" OR "risk estimation" OR "risk algorithm" OR nomogram OR "risk calculator" OR "predictive model") OR AB("risk prediction" OR "risk score" OR "risk model" OR "prognostic model" OR "prediction model" OR "risk assessment" OR "risk stratification" OR "risk estimation" OR "risk algorithm" OR nomogram OR "risk calculator" OR "predictive model")))	33
Embase	#1 search: ('malignant neoplasm'/exp OR 'malignant neoplasm') AND [embase]/lim<br>#2 search: ('cancer':ti,ab,kw OR 'cancers':ti,ab,kw OR 'malignant neoplasia':ti,ab,kw OR 'malignant neoplastic disease':ti,ab,kw OR 'malignant tumor':ti,ab,kw OR 'malignant tumour':ti,ab,kw OR 'neoplasia, malignant':ti,ab,kw OR 'neoplasmic malignancy':ti,ab,kw OR 'neoplastic malignancy':ti,ab,kw OR 'oncologic malignancy':ti,ab,kw OR 'oncological malignancy':ti,ab,kw OR 'tumor, malignant':ti,ab,kw OR 'tumoral malignancy':ti,ab,kw OR 'tumorous malignancy':ti,ab,kw OR 'tumour, malignant':ti,ab,kw OR 'malignant neoplasm':ti,ab,kw) AND [embase]/lim<br>#3 search: #1 OR #2<br>#4 search: ('postoperative delirium'/exp OR 'postoperative delirium') AND [embase]/lim<br>#5 search: ('delirium after surgery':ti,ab,kw OR 'delirium after surgical intervention':ti,ab,kw OR 'delirium after surgical repair':ti,ab,kw OR 'delirium after surgical resection':ti,ab,kw OR 'post-operative confusion':ti,ab,kw OR 'post-operative deliria':ti,ab,kw OR	23

	'post-operative delirium':ti,ab,kw OR 'postoperative confusion':ti,ab,kw OR 'postoperative deliria':ti,ab,kw OR 'postoperative delirious state':ti,ab,kw OR 'postoperative delirium':ti,ab,kw) AND [embase]/lim<br>#6 search: #4 OR #5<br>#7 search: ('predictive model'/exp OR 'predictive model') AND [embase]/lim<br>#8 search: ('forecast model':ti,ab,kw OR 'forecast modeling':ti,ab,kw OR 'forecast modelling':ti,ab,kw OR 'forecast simulation':ti,ab,kw OR 'forecasting model':ti,ab,kw OR 'prediction model':ti,ab,kw OR 'predictive modeling':ti,ab,kw OR 'predictive modelling':ti,ab,kw OR 'predictive simulation':ti,ab,kw OR 'predictive model':ti,ab,kw) AND [embase]/lim<br>#9 search: #7 OR #8<br>#10 search: #3 AND #6 AND #9	
PubMed	#1 search: ("neoplasms"[MeSH Terms] OR "carcinoma"[MeSH Terms] OR "neoplasms"[Title/Abstract] OR "carcinoma"[Title/Abstract] OR "cancer"[Title/Abstract] OR "cancer*"[Title/Abstract] OR "oncology"[Title/Abstract] OR "carcinoma*"[Title/Abstract] OR "tumor*"[Title/Abstract] OR "tumors"[Title/Abstract] OR "tumour"[Title/Abstract] OR "tumours"[Title/Abstract] OR "neoplasm"[Title/Abstract] OR "neoplasm*"[Title/Abstract] OR "malignan*"[Title/Abstract] OR "metasta*"[Title/Abstract] OR "sarcoma*"[Title/Abstract] OR "glioma*"[Title/Abstract])<br>#2 search: ("delirium"[MeSH Terms] OR "delirium*"[Title/Abstract] OR "deliri*"[Title/Abstract] OR "postoperative delirium"[Title/Abstract] OR "post operative delirium"[Title/Abstract] OR "postsurgical delirium"[Title/Abstract] OR "postoperative cognitive dysfunction"[Title/Abstract] OR "emergence delirium"[Title/Abstract] OR "acute confusion*"[Title/Abstract] OR "temporary confusion*"[Title/Abstract] OR "subacute delirium*"[Title/Abstract])<br>#3 search: ("risk prediction"[Title/Abstract] OR "risk score"[Title/Abstract] OR "risk model"[Title/Abstract] OR "prognostic model"[Title/Abstract] OR "prediction model"[Title/Abstract] OR "risk assessment"[Title/Abstract] OR "risk stratification"[Title/Abstract] OR "risk estimation"[Title/Abstract] OR "risk algorithm"[Title/Abstract] OR "nomogram"[Title/Abstract] OR "risk calculator"[Title/Abstract] OR "predictive model"[Title/Abstract])<br>#4 search: #1 and #2 and #3	88
PsycINFO	#1 search: Neoplasms.mf.<br>#1 search: (neoplasms or carcinoma or cancer* or oncology or tumor* or tumour* or malignan* or metasta* or sarcoma* or glioma*).ab,ti.<br>#3 search: #1 OR #2<br>#4 search: Delirium.mf.<br>#5 search: (delirium* or deliri* or "postoperative delirium" or "post	10

	operative delirium" or "postsurgical delirium" or "emergence delirium" or "acute confusion*" or "temporary confusion*" or "subacute delirium*" or "postoperative cognitive dysfunction").ab,ti.<br>#6 search: #4 OR #5<br>#7 search: Risk Assessment.mf.<br>#8 search: ("risk prediction" or "risk score" or "risk model" or "prediction model" or "prognostic model" or "risk stratification" or "risk estimation" or "risk algorithm" or nomogram or "risk calculator" or "predictive model").ab,ti.<br>#9 search: #7 OR #8<br>#10 search: #3 AND #6 AND #9	
Web of Science	#1 search: TS=(neoplasms OR carcinoma OR cancer* OR oncology OR tumor* OR tumour* OR malignan* OR metasta* OR sarcoma* OR glioma*)<br>#2 search: TS=(delirium* OR "postoperative delirium" OR "post operative delirium" OR "postsurgical delirium" OR "emergence delirium" OR "acute confusion*" OR "temporary confusion*" OR "subacute delirium*")<br>#3 search: TS=("risk prediction" OR "risk score" OR "risk model" OR "prognostic model" OR "prediction model" OR "risk assessment" OR "risk stratification" OR "risk estimation" OR "risk algorithm" OR nomogram OR "risk calculator" OR "predictive model")<br>#4 search: #1 AND #2 AND #3	107
CNKI	(主题：肿瘤 + 癌 + 恶性肿瘤 + 癌症) AND (主题：谵妄 + 术后谵妄 + 苏醒谵妄) AND (主题：风险预测模型 + 预测模型 + 预后模型 + 列线图 + 预测因子 + 机器学习模型)	30
WanFang	主题:(肿瘤 OR 癌 OR 恶性肿瘤 OR 癌症) and 主题:(谵妄 OR 术后谵妄 OR 苏醒谵妄) and 主题:(风险预测模型 OR 预测模型 OR 预后模型 OR 列线图 OR 预测因子 OR 机器学习模型)	61
VIP	(((((题名或关键词=肿瘤 OR 题名或关键词=癌) OR 题名或关键词=恶性肿瘤) OR 题名或关键词=癌症) AND ((题名或关键词=谵妄 OR 题名或关键词=术后谵妄) OR 题名或关键词=苏醒谵妄)) AND (((((题名或关键词=风险预测模型 OR 题名或关键词=预测模型) OR 题名或关键词=预后模型) OR 题名或关键词=列线图) OR 题名或关键词=预测因子) OR 题名或关键词=机器学习模型))	15
CBM	#1："肿瘤"[常用字段:智能] OR "癌"[常用字段:智能] OR "恶性肿瘤"[常用字段:智能] OR "癌症"[常用字段:智能]<br>#2："谵妄"[常用字段:智能] OR "术后谵妄"[常用字段:智能] OR "苏醒谵妄"[常用字段:智能]<br>#3："风险预测模型"[常用字段:智能] OR "预测模型"[常用字段:智能] OR "预后模型"[常用字段:智能] OR "列线图"[常用字段:智能] OR "预测因子"[常用字段:智能] OR "机器学习模型"[常用字段:智能]<br>#4：(#3) AND (#2) AND (#1)	78

Table S2. Assessment of potential cohort overlap or data reuse among included studies.

Potentially related studies	Reason for checking	Key information compared	Judgment	Handling in this review
Wan et al. (2025) [10] and Zhao et al. (2024) [46]	Shared research group and highly similar data source	Both studies were conducted by the Southwest Medical University research group and included patients undergoing colorectal cancer surgery in 2023. Both reported a sample size of 555 patients and the same clinical trial registration number, ChiCTR2300075723.	Clear or highly likely cohort overlap	Both studies were retained because they addressed different modeling or analytical aims. However, they were interpreted as related analyses rather than fully independent cohorts.
Xiang et al. (2023) [16] and Zhu et al. (2025) [15]	Shared authorship and institution	Both studies involved the Taizhou People's Hospital/Nanjing Medical University research group and shared authors, including Dong Xiang, Hailin Xing, and Yabin Zhu. Xiang et al. [16] included elderly patients undergoing laparoscopic surgery for gynecologic cancers from March 2017 to May 2021, whereas Zhu et al. [15] included geriatric patients undergoing radical	Possible partial overlap	Both studies were retained, but the possibility of partial cohort overlap was flagged. Their findings were interpreted cautiously, especially when considering evidence independence.

		hysterectomy for cervical cancer from 2021 to 2025.		
Oral/head and neck cancer or free flap-related studies, including Shen and Wang (2025) [25], Shen et al. (2023) [26], Chu et al. (2025) [29], Chen et al. (2025b) [12], and Choi et al. (2017) [11]	Similar clinical topic	These studies focused on oral cancer, head and neck cancer, or free flap reconstruction. However, available information indicated differences in author groups, institutions, countries, study periods, sample sizes, or clinical settings.	No clear evidence of cohort overlap	These studies were treated as independent cohorts based on available information.
Zhu et al. (2026) [9]	Use of public and synthetic data	This study used a public real-world dataset and synthetic data for model development or evaluation. The independence of the original public dataset from all other included clinical cohorts could not be fully verified from the available information.	Unclear independence of original public dataset	The study was retained as a secondary data/modeling study, but its findings were interpreted cautiously.
Other included studies	General check for overlap	Other included studies, including studies with similar author-year labels, were checked by comparing	No clear evidence of cohort overlap based on available information	These studies were treated as independent evidence in the narrative synthesis.

authorship, institutions, study periods, sample sizes, cancer types, surgical procedures, and data sources. No clear evidence of cohort overlap was identified based on available information.

Table S3. Diagnostic criteria for POD in included studies (n=32).

Authors/Years/Country	Diagnosis criteria of POD
Choi et al. (2017) [11], South Korea	DSM-IV
Flanigan et al. (2018) [13], USA	DSM-V
Mosk et al. (2018) [42], the Netherlands	DSM-IV
Yajima et al. (2023) [14], Japan	CAM/DSM-V
Shen and Xu (2024) [24], China	3D-CAM/ICU-CAM
Shen and Wang (2025) [25], China	CAM
Shen et al. (2023) [26], China	CAM
Chen et al. (2026) [27], China	CAM/CAM-ICU
Chen et al. (2023) [28], China	CAM
Chu et al. (2025) [29], China	4AT
Gao and Huan (2025) [30], China	CAM-ICU
Li et al. (2022) [31], China	CAM
Liang and Xie (2023) [32], China	CAM
Tang et al. (2025) [33], China	CAM
Weng et al. (2024) [34], China	CAM
Xu et al. (2025) [35], China	CAM
Ye et al. (2022) [36], China	CAM
Zhang et al. (2023) [37], China	CAM
Zhang et al. (2025) [38], China	CAM
Chen et al. (2025a) [39], China	CAM
Chen et al. (2025b) [12], China	CAM-ICU
Hu et al. (2024) [40], China	CAM
Liu et al. (2023) [41], China	CAM-ICU
Shen et al. (2025) [43], China	ASER/POQI
Wan et al. (2025) [10], China	3D-CAM
Wang et al. (2025) [44], China	3D-CAM
Xiang et al. (2023) [16], China	DSM-V
Xue et al. (2025) [17], China	DSM-IV
Yan et al. (2024) [45], China	CAM
Zhao et al. (2024) [46], China	3D-CAM
Zhu et al. (2026) [9], China	DSM-V
Zhu et al. (2025) [15], China	CAM

Abbreviations: 3-Minute Diagnostic Interview for CAM-defined Delirium (3D-CAM), Confusion Assessment Method (CAM), Confusion Assessment Method for the Intensive Care Unit (CAM-ICU), Diagnostic and Statistical Manual of Mental Disorders, Fifth Edition (DSM-5), Diagnostic and Statistical Manual of Mental Disorders, Fourth Edition (DSM-IV), Four-item

Acute Delirium Test (4AT), Perioperative Quality Improvement Institute (POQI), American Society for Enhanced Recovery (ASER).

Table S4. Comparative summary of machine-learning-based POD prediction models.

**Item**	**Shen and Xu (2024)** [24]**, China**	**Weng et al. (2024)** [34]**, China**	**Wan et al. (2025)** [10]**, China**	**Zhu et al. (2026)** [9]**, China**
Cancer type / surgery	Esophageal cancer; thoracoscopic radical surgery	Urological oncology surgery in elderly patients	Colorectal cancer; radical surgery	Lung cancer; lung cancer resection
Dataset and imbalance	n=194; POD=90/194 (46.39%). Class imbalance was not prominent.	n=1,180; overall POD incidence=9.40%, indicating class imbalance. POD incidence was 9.1% in the modeling cohort and 10.2% in the validation cohort.	n=555; POD=100/555 (18.02%), indicating moderate class imbalance.	Synthetic training set n=2,000; real-world test set n=570; total n=2,570. POD incidence=6.70%, indicating substantial class imbalance.
Class imbalance handling	No specific imbalance-handling method was reported.	No specific resampling, class weighting, or other imbalance-handling method was reported.	No specific imbalance-handling method was reported.	Synthetic Data Vault (SDV) was used to generate synthetic data, but explicit class weighting or resampling for imbalance was not clearly reported.
Feature selection strategy	Univariate analysis and multivariable logistic regression were used to identify POD-related risk factors before CART model construction.	LASSO regression with cross-validation was used to select predictors. Five predictors were retained: age, diabetes, ASA grade, preoperative albumin, and operation time.	LASSO regression with cross-validation retained 12 predictive features. SHAP was further used to identify important predictors for the final model and online tool.	Clinically relevant variables were selected. Variables with >30% missing data were excluded. Missing values were imputed, outliers were handled by Winsorization, and continuous variables were normalized. Key predictors included preoperative blood glucose, vital capacity, mean corpuscular volume, and preoperative albumin.
Machine-learning algorithm(s)	Classification and regression tree (CART) decision tree; logistic regression was used as a comparator.	Logistic regression, random forest, support vector machine, extreme gradient boosting, artificial neural network, and Bayesian network.	Logistic regression, support vector machine, gradient boosting machine, neural network, random forest, XGBoost, K-nearest neighbors, AdaBoost, LightGBM, and	Logistic regression, decision tree, random forest, gradient boosting decision tree, XGBoost, LightGBM, CatBoost, linear support vector machine, multilayer perceptron, Gaussian Naive Bayes, K-nearest neighbors, and AdaBoost.

			CatBoost.	
Validation method	Not clearly reported; model performance was mainly assessed in the study dataset.	Random split at a 7:3 ratio into modeling and validation cohorts.	Random split into training and validation sets at a 60:40 ratio; five-fold cross-validation was applied.	Synthetic data were used as the training set, and real-world Figshare data were used as the test set; five-fold cross-validation was reported.
Reported performance metrics	CART model: AUC=0.837 (95% CI: 0.777–0.886). Logistic regression model: AUC=0.777 (95% CI: 0.712–0.834). Accuracy, sensitivity, specificity, and calibration were not fully reported.	Random forest showed the best performance. Development cohort: AUC=0.889, accuracy=0.817, sensitivity=0.893, specificity=0.699. Validation cohort: AUC=0.882, accuracy=0.801, sensitivity=0.941, specificity=0.746.	AUC values ranged from 0.708 to 0.802 across models. SVM had the highest AUROC value of 0.802 (95% CI: 0.705–0.898), while random forest had an AUROC of 0.795 (95% CI: 0.704–0.885) and was selected as the final model based on decision curve analysis. Accuracy, sensitivity, and specificity were not consistently reported in the extracted results.	LinearSVC achieved the highest AUC of 0.763, followed by logistic regression (AUC=0.754), multilayer perceptron classifier (AUC=0.752), and Gaussian Naive Bayes (AUC=0.727). Gaussian Naive Bayes had an accuracy of 89.8%, recall of 0.263, and F1-score of 0.256.
Interpretability / methodological comments	The CART model is relatively intuitive and clinically interpretable. However, the study was based on a single-center dataset, lacked external validation, and compared only CART with logistic regression.	This study compared multiple algorithms and reported several performance metrics. SHAP was used to visualize feature importance. However, the low POD incidence indicates class imbalance, and external validation was not performed.	This study compared a broad range of machine-learning algorithms and used SHAP, decision curve analysis, mediation analysis, and an online Shiny-based prediction tool. However, external validation was limited, and the cohort may overlap with another included study;	This study compared the largest number of artificial intelligence algorithms and used LIME for local interpretability. However, the use of synthetic data, low POD incidence, low recall despite high accuracy, and unclear independence of the public real-world dataset limit clinical generalizability.

			therefore, the independence of evidence should be interpreted cautiously.

Abbreviations: adaptive boosting (AdaBoost); American Society of Anesthesiologists physical status classification (ASA); area under the receiver operating characteristic curve (AUC); classification and regression tree (CART); decision curve analysis (DCA); gradient boosting decision tree (GBDT); least absolute shrinkage and selection operator (LASSO); light gradient boosting machine (LightGBM); Local Interpretable Model-agnostic Explanation (LIME); machine learning (ML); mean corpuscular volume (MCV); not reported (NR); postoperative delirium (POD); Shapley Additive Explanations (SHAP); Synthetic Data Vault (SDV); support vector machine (SVM); vital capacity (VC); extreme gradient boosting (XGBoost).

Note: Only studies that used machine-learning or artificial intelligence algorithms as prediction models were included in this table. Studies that used LASSO only for variable selection but constructed final models using logistic regression or nomograms were not classified as machine-learning-based prediction model studies in this table.

Table S5. Key reporting characteristics of included prediction model studies according to selected TRIPOD-relevant items (n=32).

Authors/Years/Country	Outcome reporting		Predictor reporting		Sample and data handling		Model specification and validation					Model presentation
	POD assessment method	POD assessment window	Candidate predictor definition	Predictor selection strategy	Number of POD events	Missing data handling	Complete model specification	Internal validation	External validation	Calibration assessment	Clinical utility evaluation	Model presentation /availability
Choi et al. (2017) [11], South Korea	Yes	Partial	Partial	Yes	Yes	Partial	Partial	Yes	No	No	No	Yes/Partial
Flanigan et al. (2018) [13], USA	Yes	Yes	Partial	Yes	Yes	NR	Yes	No	Yes	Partial	No	Yes/Partial
Mosk et al. (2018) [42], the Netherlands	Yes	Yes	Yes	Yes	Yes	Partial	Partial	No	No	Partial	No	Yes/Partial
Yajima et al. (2023) [14], Japan	Yes	Yes	Yes	Yes	Yes	Partial	Partial	No	Yes	Yes	No	Partial
Shen and Xu (2024) [24], China	Yes	Yes	Partial	Yes	Yes	Partial	Partial	No	No	No	No	Yes/Partial
Shen and Wang (2025) [25], China	Yes	Yes	Partial	Yes	Yes	Partial	Partial	Yes	No	Yes	No	Yes/Partial
Shen et al. (2023) [26], China	Yes	Yes	Partial	Yes	Yes	NR	Partial	No	No	No	No	Partial
Chen et al. (2026) [27], China	Yes	Yes	Yes	Yes	Yes	Partial	Partial	Yes	No	Yes	Yes	Yes/Partial
Chen et al. (2023) [28], China	Yes	Yes	Partial	Yes	Yes	Partial	Yes	Yes	Yes	Yes	Yes	Yes/Partial
Chu et al. (2025) [29],	Yes	Yes	Partial	Yes	Yes	Partial	Partial	Yes	No	Yes	Yes	Yes/Partial

China												
Gao and Huan (2025) [30], China	Yes	Yes	Partial	Yes	Yes	Partial	Partial	Partial	No	Partial	Yes	Yes/Partial
Li et al. (2022) [31], China	Yes	Yes	Partial	Yes	Yes	Partial	Partial	Yes	No	Yes	Yes	Yes/Partial
Liang and Xie (2023) [32], China	Yes	Yes	Partial	Yes	Yes	NR	Partial	Yes	No	Yes	Yes	Yes/Partial
Tang et al. (2025) [33], China	Yes	Yes	Partial	Yes	Yes	Partial	Partial	Partial	Yes	Yes	Yes	Yes/Partial
Weng et al. (2024) [34], China	Yes	Yes	Partial	Yes	Partial	Partial	Partial	Yes	No	No	No	Yes/Partial
Xu et al. (2025) [35], China	Yes	Yes	Partial	Yes	Yes	Partial	Partial	Partial	Yes	Yes	No	Yes/Partial
Ye et al. (2022) [36], China	Yes	Yes	Partial	Yes	Yes	NR	Yes	No	No	Yes	No	Partial
Zhang et al. (2023) [37], China	Yes	Yes	Partial	Yes	Partial	Partial	Partial	Partial	Yes	Yes	No	Yes/Partial
Zhang et al. (2025) [38], China	Yes	Yes	Partial	Yes	Yes	NR	Yes	Yes	No	No	No	Yes/Partial
Chen et al. (2025a) [39], China	Yes	Yes	Partial	Yes	Yes	Partial	Partial	No	No	Yes	Yes	Yes/Partial
Chen et al. (2025b) [12], China	Yes	Yes	Partial	Yes	Yes	Partial	Partial	Yes	No	Yes	Yes	Yes/Partial
Hu et al. (2024) [40], China	Yes	Yes	Partial	Yes	Yes	NR	Partial	Partial	No	Yes	No	Partial
Liu et al. (2023) [41],	Yes	Yes	Partial	Yes	Yes	Partial	Partial	No	No	No	No	Yes/Partial

China												
Shen et al. (2025) [43], China	Partial	Partial	Yes	Yes	Yes	Partial	Partial	Yes	No	Yes	Yes	Yes/Partial
Wan et al. (2025) [10], China	Yes	Yes	Yes	Yes	Yes	Yes	Yes	Yes	No	No	Yes	Yes
Wang et al. (2025) [44], China	Yes	Yes	Yes	Yes	Yes	Partial	Partial	Yes	No	Yes	Yes	Yes/Partial
Xiang et al. (2023) [16], China	Yes	Yes	Partial	Yes	Yes	Partial	Partial	No	No	Yes	Yes	Yes/Partial
Xue et al. (2025) [17], China	Yes	Yes	Partial	Yes	Yes	Partial	Partial	Yes	Yes	Yes	No	Yes/Partial
Yan et al. (2024) [45], China	Yes	Yes	Partial	Yes	Yes	Partial	Partial	No	Yes	Yes	Yes	Yes/Partial
Zhao et al. (2024) [46], China	Yes	Yes	Partial	Yes	Partial	Partial	Partial	No	No	Yes	No	Partial
Zhu et al. (2026) [9], China	Partial	Partial	Partial	Yes	Partial	Partial	Partial	Yes	No	No	No	Yes/Partial
Zhu et al. (2025) [15], China	Yes	Yes	Partial	Yes	Yes	Partial	Partial	Yes	Yes	Yes	Yes	Yes/Partial

Abbreviations: not reported (NR).

Definitions: Yes, the item was clearly reported; No, the item was not performed or not provided; Partial, the item was incompletely reported (e.g., logistic regression mentioned but coefficients or intercept not fully presented).

Table S6. Frequency of the most commonly reported predictor domains across included studies.

Predictor domain	Harmonized terms included	Number of studies, n (%)
Age	Age, advanced age, age defined by different cut-off values	24 (75.00)
Operation/anesthesia time	Operation time, operative time, duration of surgery, anesthesia time, extraction time, PACU stay, ICU stay, duration of mechanical ventilation, time to recovery from anesthesia	15 (46.88)
Preoperative nutritional indicators	PNI, albumin, AFR, CONUT, GNRI, preoperative nutrition risk, malnourishment, diet-related score	15 (46.88)
Preoperative inflammatory indicators	NLR, SII, SIINI, CRP, IL-1β, IL-6, lymphocyte-related indicators, PWR	9 (28.13)
American Society of Anesthesiologists classification	ASA classification, ASA grade, ASA physical status classification	6 (18.75)

Abbreviations: albumin-to-fibrinogen ratio (AFR); American Society of Anesthesiologists physical status classification (ASA); Controlling Nutritional Status (CONUT); C-reactive protein (CRP); Geriatric Nutritional Risk Index (GNRI); intensive care unit (ICU); interleukin (IL); neutrophil-to-lymphocyte ratio (NLR); post-anesthesia care unit (PACU); prognostic nutritional index (PNI); platelet-to-white blood cell ratio (PWR); systemic immune-inflammation index (SII); systemic immune-inflammatory-nutritional index (SIINI).

Note: Predictor frequencies were counted at the study level. Similar predictor terms were harmonized before counting. When the same predictor appeared in multiple models within one study, it was counted only once. Percentages were calculated using the total number of included studies as the denominator (n =32).
